# Magnetic resonance-guided laser interstitial thermal therapy vs. stereoelectroencephalography-guided radiofrequency thermocoagulation in epilepsy patients with focal cortical dysplasia: a systematic review and meta-analysis

**DOI:** 10.3389/fneur.2023.1241763

**Published:** 2023-10-20

**Authors:** Yiming Li, Jiayi Gao, Zi Ye, Jie Mu

**Affiliations:** ^1^West China Clinical Medical School, West China Hospital, Sichuan University, Chengdu, China; ^2^Department of Neurology, West China Hospital, Sichuan University, Chengdu, China

**Keywords:** magnetic resonance-guided laser interstitial thermal therapy, stereoelectroencephalography-guided radiofrequency thermocoagulation, focal cortical dysplasia, seizure, seizure-free

## Abstract

**Background:**

Magnetic resonance-guided laser interstitial thermal therapy (MRgLiTT) and stereoelectroencephalography-guided radiofrequency thermocoagulation (SEEG-RFTC) are two effective, minimally invasive treatments for epilepsy with focal cortical dysplasia (FCD). The purpose of this study is to conduct a meta-analysis to evaluate and compare the efficacy and safety of these two therapies in epilepsy patients with FCD.

**Methods:**

We searched PubMed, Embase, Cochrane, and other databases for articles published before March 2023. The primary objective was to compare the effectiveness and complications of MRgLiTT and SEEG-RFTC in epilepsy patients with FCD. The second objective was to determine which method provides a better prognosis for specific subgroup patients.

**Results:**

According to the inclusion and exclusion criteria, 18 studies were included, comprising 270 FCD patients including 37 patients from 6 MRgLiTT studies and 233 from 12 SEEG-RFTC studies. There were no significant differences between MRgLiTT and SEEG-RFTC groups in the seizure-freedom rate (59%, 95% CI 44–74%; 52%, 95% CI 47–57%, *P* = 0.86) and the rate of ≥50% seizure-reduction of FCD (90%, 95% CI 80–100%; 90%, 95% CI 86–94%, *P* = 0.42). Both methods had low complication rates (17.1%, 28/159) and long-term complication (2.5%, 4/159) rate, with no significant difference between them (*P* = 0.17).

**Conclusion:**

Both MRgLiTT and SEEG-RFTC are safe and minimally invasive treatments for patients with FCD. They have comparable performance in terms of postoperative seizure-freedom rates in patients with FCD, and both can be used as treatment options for patients with FCD. Our study found that SEEG-RFTC had a better therapeutic effect in the FCD2b subgroup.

## 1. Introduction

Focal cortical dysplasia (FCD) is one of the most common causes of refractory epilepsy (1). Surgical excision of epileptogenic foci is an effective treatment for drug-resistant epilepsy, but only approximately one-third of patients are suitable for it due to the lack of accurate mapping of the epileptogenic zone, the location of foci in functional areas, and the presence of multiple epileptogenic zones. In recent years, stereoelectroencephalography-guided radiofrequency thermocoagulation (SEEG-RFTC) and magnetic resonance-guided laser interstitial thermal therapy (MRgLiTT) have attracted much attention for their minimally invasive nature, safety, and effectiveness (2–4). In 2004, RFTC guided by stereotactic electroencephalogram (SEEG) recording was reported to be safe and led to a significant reduction in seizure frequency (5, 6). MRgLiTT is guided by real-time MRI to selectively ablate lesions or structures using heat released by laser to treat a variety of intracranial lesions (7). MRgLiTT, regarded as a less invasive alternative to open surgery, is more precise and predictable in terms of tissue ablation volume than other techniques for local tissue ablation, resulting in less collateral damage (8, 9).

The two therapies have been well established in the treatment of various etiologies (6, 10), but it remains unknown which method is superior for FCD patients. In this meta-analysis, we address this question by comparing the seizure-freedom rate and postoperative complications between MRgLiTT and SEEG-RFTC in patients with FCD.

## 2. Methods

### 2.1. Search strategy and inclusion criteria

This study was conducted according to the preferred reporting item of the Systematic Evaluation and Meta-Analysis (PRISMA) guidelines (see the PRISMA checklist) (11). A comprehensive search was made in databases including PubMed, Embase, and Cochrane using the following terms (adding an appendix “[Title/Abstract]” to each term): stereo-electroencephalography, radiofrequency-thermocoagulation, thermo-SEEG, laser ablation, laser interstitial thermotherapy, and focal cortical dysplasia. The search ended in March 2023.

Although pathologic confirmation is the gold standard for the diagnosis of FCD, the results of the pathology report were not available because all patients received non-excision treatment. We usually make a diagnosis based on seizure characteristics (drug-refractory epilepsy with a previous diagnosis of focal seizures and recent seizures), FCD imaging features (focal cortical thickening, poorly demarcated gray and white matter, cortical and/or subcortical white matter, and T2WI/FLAIR high signal, etc.), and electroencephalographic features of FCD (rhythmic epileptiform discharges, RED). The determination of FCD in this article was based on clinical experience (drug-resistance epilepsy; MRI characteristics of the lesions; EEG shows focal rhythmic interictal epileptiform discharges that may correlate spatially with the anatomic extent of the lesion) by the authors of the cited article, and the subtypes of the FCD were also classified by the original authors according to the pathological criteria published by the International League Against Epilepsy (ILAE) in 2011 (12), in the absence of any histopathologic data. The inclusion criteria were as follows: (1) prospective or retrospective study reporting the efficacy of SEEG-RFTC or MRgLiTT in patients with FCD; (2) providing detailed information of seizure-free patients and complications; (3) more than 1 month follow-up time.

The exclusion criteria were as follows: (1) a summary or abstract without full text; (2) MRgLiTT and/or SEEG-RFTC performed as a secondary choice after the failure of a prior operation; and (3) population overlap (when there was a duplication in the inclusion populations of two articles, the earlier published study was removed); (4) case reports.

### 2.2. Data extraction and assessment

Two investigators worked independently on study selection, data extraction, and quality assessment (YML and JYG). To assess the quality of the search strategy, both investigators screened the titles and abstracts of articles. Disagreement was settled through consultation with the senior author (JM). Full-text versions of all eligible studies were used for quality assessment (risk of bias) and data collection. The following information was extracted from each study: first author, year of publication, country and center, surgical period, study design, sample size, gender, age range, duration of follow-up, Engel classification, and complication. Data were calculated separately by the two investigators using standard extraction rules. In addition, the risk of bias in the included studies was assessed using the MINOR (methodological index for non-randomized studies) tool (10).

The outcomes of MRgLiTT and SEEG-RFTC procedures were rated in three classes: Class 1. seizure-free, i.e., no seizure attack after the coagulation, equal to the Engle class I; Class 2. responding, i.e., patients did not develop into seizure-free but had ≥50% improvement of epilepsy, equal to the Engle classes II and III; and Class 3. non-responding, i.e., <50% improvement of epilepsy or no improvement or even becoming worse, equal to the Engle class IV. In articles using the ILAE classification, ILAE1 was considered equal to Engel 1, indicative of seizure freedom. In the analysis of complications, all postoperative complications were divided into transient and permanent complications (defined as a complication that did not resolve at the time of discharge) for separate analyses as different types of complications do not affect patients to the same degree.

### 2.3. Statistical analyses

Raw proportions were used to calculate prevalence and 95% confidence interval (CI). The *I*^2^ index was used to assess heterogeneity. If the *I*^2^ index is >50% or the *P*-value is < 0.05, heterogeneity is considered to be significant, and the random effect model will be used. Otherwise, the fixed effects model would be used. For each study, forest plots were created to show a 95% CI of prevalence. Subgroup analyses were performed to investigate clinical heterogeneity. The funnel plot was used to assess potential publication bias, and Egger's linear regression test was used to determine whether the publication bias was statistically significant (13). A *P*-value of < 0.05 was considered to be statistically significant (two-sided). R statistical (version 4.1.1) software was used for all analyses.

## 3. Results

### 3.1. Study selection and quality assessment

**Figure 1:**
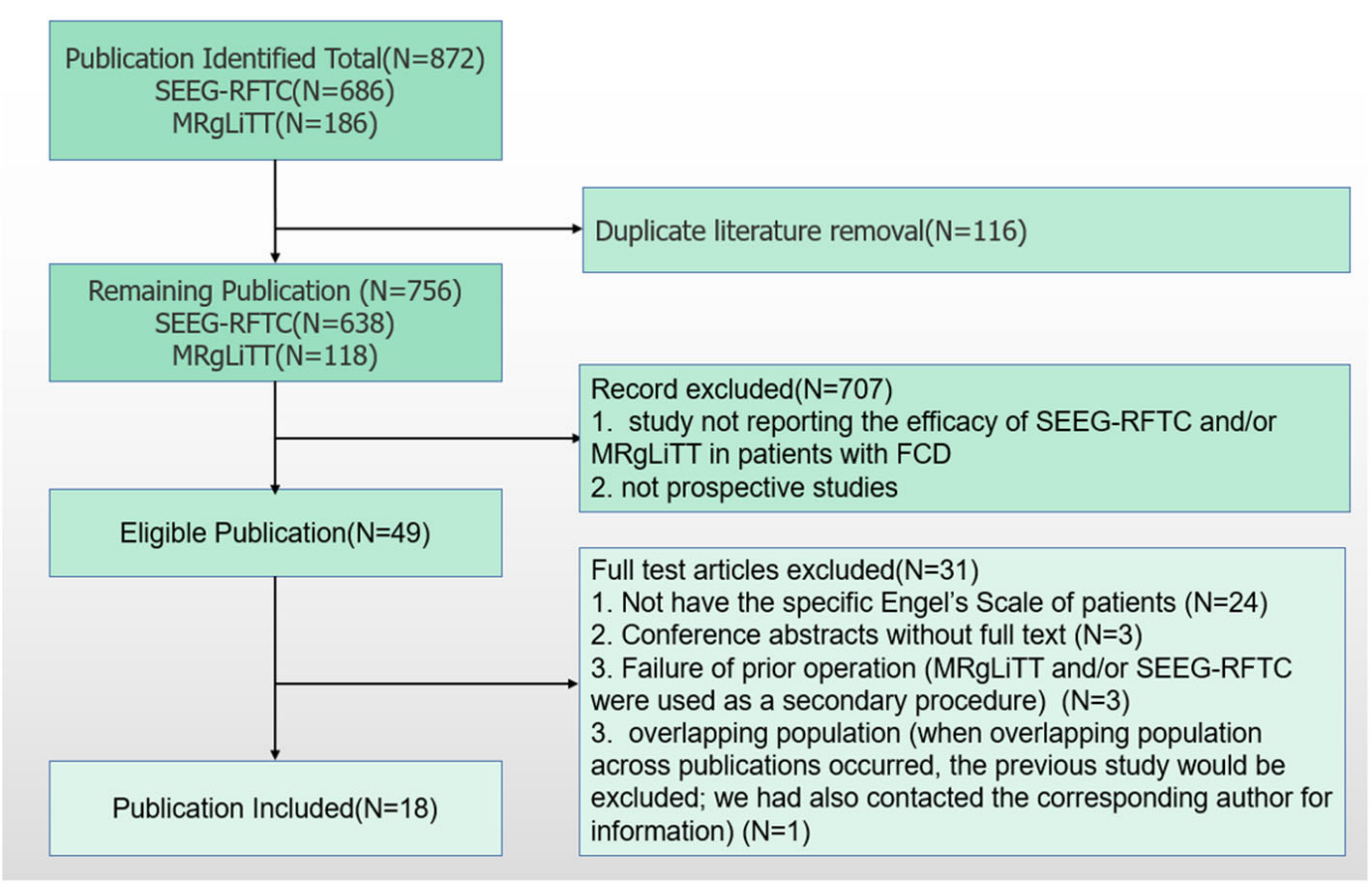
Diagram of systematic search.

According to the search strategy, 872 articles were obtained from the online databases. After abstract screening and duplicate removal, 49 articles were remaining. By full-text reviewing, 24 articles were further excluded as they did not provide specific Engel classifications of patients, and three articles were conference abstracts without full-text. Three studies employed patients receiving MRgLiTT and/or SEEG-RFTC as a secondary procedure after failure to respond to a prior operation (14–16). Three articles used the same basic data, of which the study with the largest time span of patient enrollment was used in this study (17–19). At last, 14 articles were included for meta-analysis, including 6 for MRgLiTT (20–25) and 12 for SEEG-RFTC (15, 17, 19, 26–34). The flow chart of this study is shown in Figure 1.

### 3.2. Population characteristics

The total number of FCD patients was 270, with 37 (13.7%) in the MRgLiTT group and 233 (86.3%) in the SEEG-RFTC group. Table 1 shows the essential characteristics of the 14 studies.

**Table 1:** Clinical characteristics and outcomes in included studies.

**References**	**Country (center)**	**Surgical period**	**Study design**	**Sample size (*n*), male**	**Mean age (range), years**	**Mean follow-up (range), months**		**Engel class**				**Patients with post-operative complications**		
								**1**	**2**	**3**	**4**	**Transient complications**	**Long-term complications**	**MINORs**
**SEEG-RFTC**														
Bourdillon et al. (26)	France (Pierre Wertheimer Neurological and Neurosurgical Hospital, Lyon University)	2003–2013	Prospective; no compare; single center	40	-	-	FCD	6	6	10	18	2		16
Dimova et al. (29)	France (University Grenoble Alpes and Michallon Hospital, Grenoble)	2000–2014	Retrospective; no compare; single center	4 (3)	19.3 ± 8.9 (6–25)	48.1 (3.5–84)	FCD	3			1	-		13
							FCD1	2						
							FCD2b	1			1			
Cossu et al. (28)	Italy (Niguarda Hospital and University of Parma)	2008–2013	Retrospective; no compare; single center	11	-	-	FCD		1		10	2		12
Guo et al. (30)	China (Guangdong Sanjiu Brain Hospital and South China Normal University)	2017–2020	Retrospective; no compare; single center	22	15 ± 9 (2–30)	20 ± 10 (6–42)	FCD	17		5		5		11
Wellmer et al. (17)	Germany (University Hospital Knappschaftskrankenhaus)	2012–2016	One center prospective (Magdeburg *n* = 3), the other retrospective (Bochum *n* = 4); no compare; two centers	7	40 ± 10.0 (29–56)	39.7 ± 15.3 (16–57)	FCD2b	5	1	1		2		14
Mirza et al. (35)	Canada and USA (McGill University and University of Kentucky)	2016–2019	Retrospective; no compare; single center	4 (1)	33.3 ± 7.1 (26–43)	22.5 ± 5.2 (16–30)	FCD	3			1	0		13
Zhao et al. (36)	China (Children's Hospital of Fudan University)	2014–2017	Retrospective; no compare; single center	13 (6)	9.1 ± 2.9 (5.5–13)	-	FCD	10	1	1	1	2	2	13
							FCD1	1	1					
							FCD2a	5			1			
							FCD2b	4		1				
Gao et al. (37)	China (Xuanwu Hospital)	2016–2019	Retrospective; no compare; single center	10 (4)	21.1 ± 11.2 (10–48)	-	FCD	8	1	1		0		11
							FCD1	2		1				
							FCD2a	3	1					
							FCD IIId	3						
Mullatti et al. (38)	UK (King's College Hospital)	-	Retrospective; no compare; single center	11 (7)	24.4 ± 10.6 (7–40)	65.4 ± 40.1 (24–144)	FCD	8	2	1		2	0	12
Deng et al. (34)	China (Beijing Children's Hospital Affiliated to Capital Medical University)	2017–2018	Retrospective; no compare; single center	21	-	6	FCD	18	0	0	3			13
Xu et al. (32)	China (Zhejiang Provincial People's Hospital)	2017–2021	Retrospective; no compare; single center	11 (9)	23.3 ± 9.7 (14–48)	21.8 ± 9.6 (8–39)	FCD	5	3	2	1	7		12
							FCD1	1			1			
							FCD2a	2	2	2				
							FCD2b	2	1					
Piergiorgio et al. [2023]	Italy (Azienda Socio-Sanitaria Territoriale Grande Ospedale Metropolitano Niguarda)	1996–2020	Retrospective; no compare; single center	79	-	-	FCD	50	29			-		16
							FCD1	9	13					
							FCD2a	1	4					
							FCD2b	40	12					
**MRgLiTT**														
Chen et al. (21)	China (Xuanwu Hospital and Capital Medical University)	2020–2021	Retrospective; no compare; single center	7	-	-	FCD	4		3		2		12
Fayed et al. (22)	USA (MedStar Georgetown University Hospital)	2013–2017	Retrospective; no compare; single center	2 (1)	16.5 (21,12)	1	FCD	1		1		0		10
Lewis et al. (23)	USA (Miami Children's Hospital)	2011–2014	Retrospective; no compare; single center	12 (4)	16.2 ± 2.9 (11–20)	20.3 ± 8.0 (9–30)	FCD	5	1	3	3	5		13
Brown et al. [2020]	USA (University of Colorado Hospital)	-	Retrospective; compare; single center	4	-	-	FCD	2	2			2	0	13
Perry et al. (24)	USA (Dell Children's Hospital)	2013–2016	Retrospective; no compare; single center	3	10.1 ± 2.2 (8–13.1)	25.3 ± 7.8 (16–35)	FCD	2			1	-		13
Hu et al. (25)	China (Beijing Tiantan Hospital)	-	Prospective; no compare; single center	9	-	1	FCD	7	0	0	2	0		12

### 3.3. Efficacy

#### 3.3.1. FCD control by MRgLiTT and SEEG-RFTC

**Figure 2:**
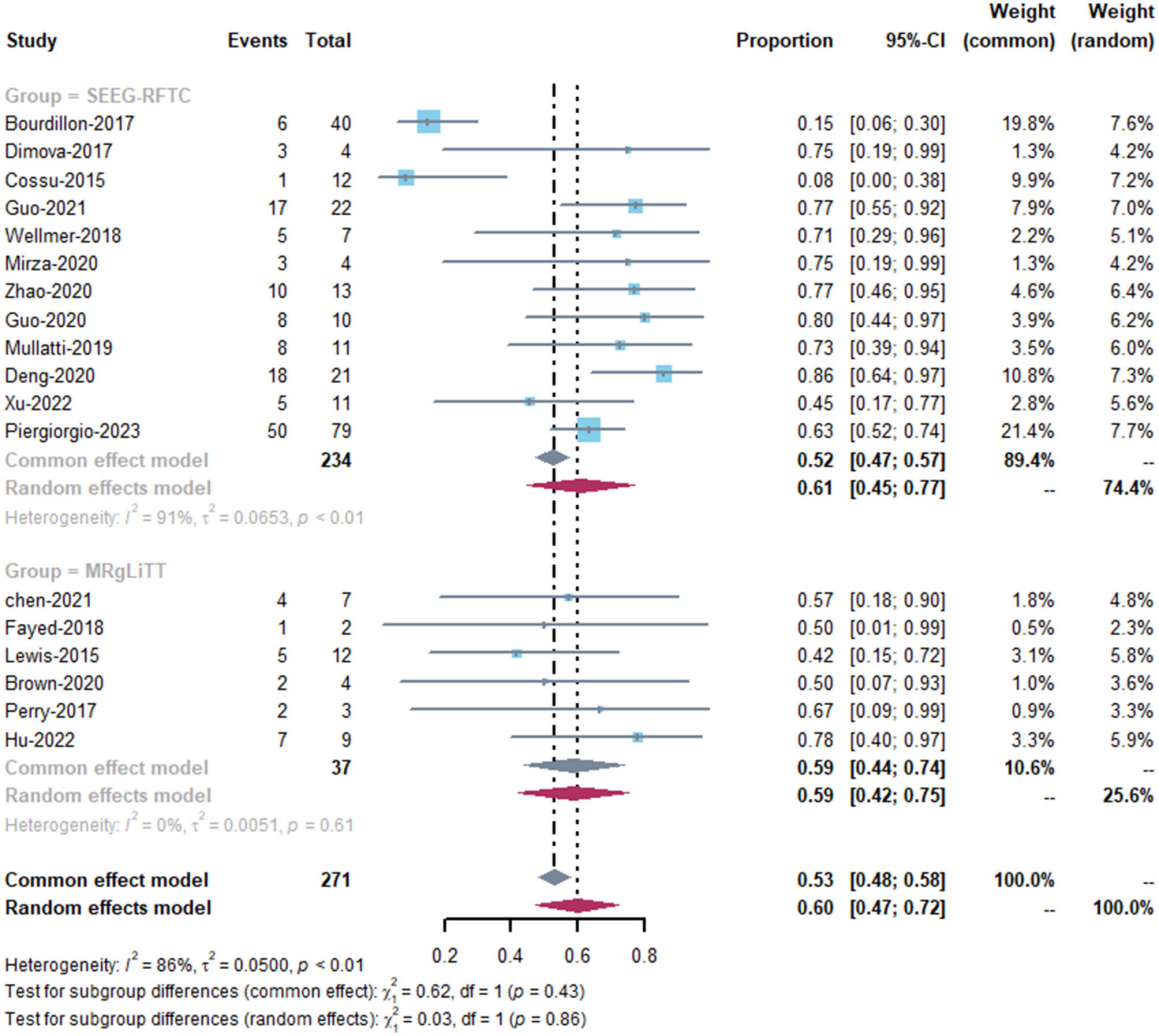
Seizure-free rate of MRgLiTT and SEEG-RFTC.

**Figure 3:**
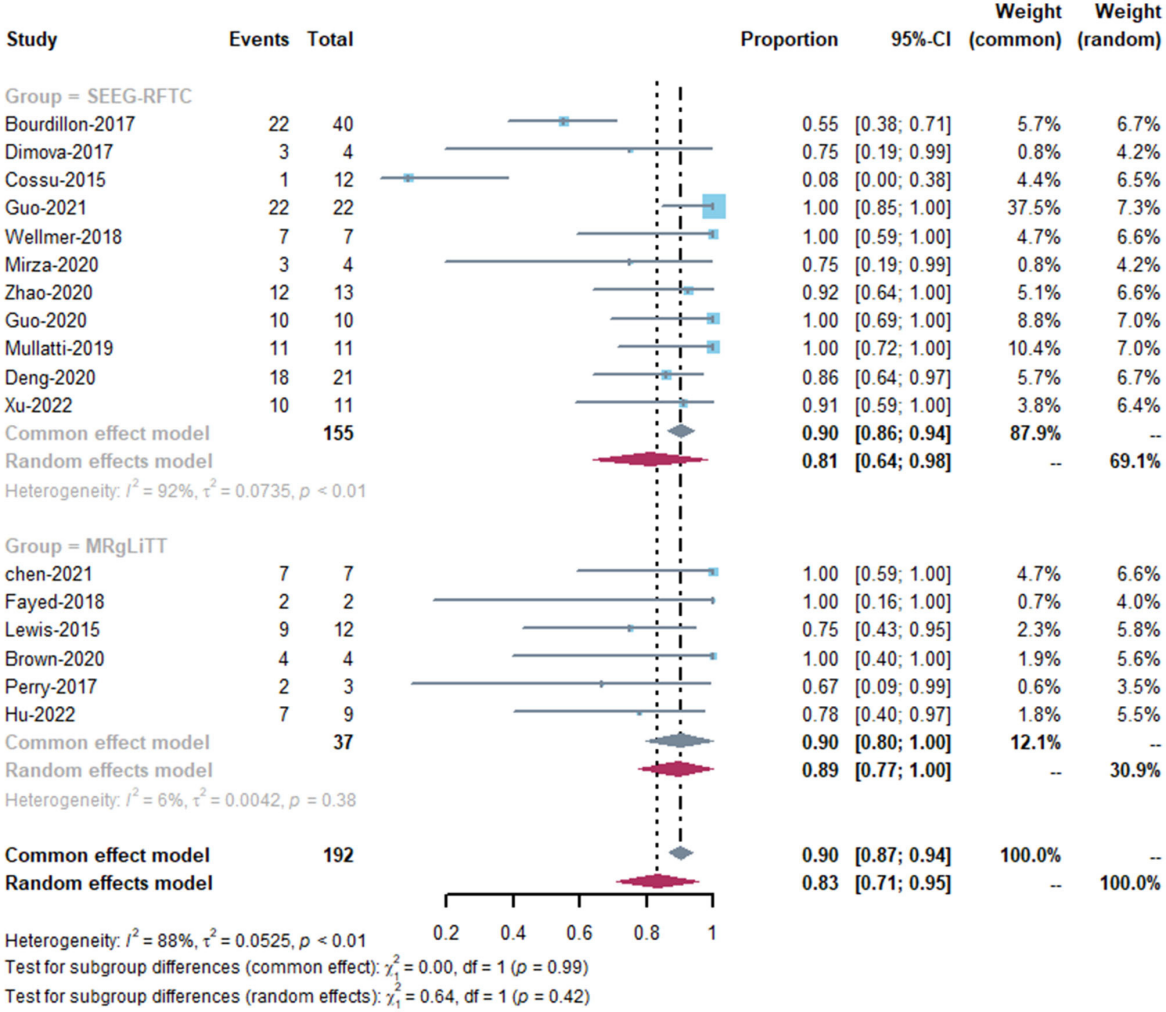
≥50% Seizure reduction rate of MRgLiTT and SEEG-RFTC.

The MRgLiTT group had a seizure-freedom rate of 42 to 78%, whereas the SEEG-RFTC group had a rate of 8 to 86%. The SEEG-RFTC group had a high heterogeneity (91%), while the MRgLiTT group had a low heterogeneity (0%). The average seizure-freedom rate in the MRgLiTT group was 59% (95% CI 44–74%), while that in the SEEG-RFTC group was 52% (95% CI 47–57%), with no significant difference between the two groups (*P* = 0.86) (Figure 2). The average rate of ≥50% seizure reduction in the MRgLiTT group was 90% (95% CI 80–100%) and that in the SEEG-RFTC group was 90% (95% CI 86–94%), with no significant difference between the two groups (*P* = 0.42) (Figure 3).

Regarding the publication bias, the funnel plots showed obvious heterogeneity among studies (Supplementary Figures 1, 2), and the Egger's test showed no statistical significance for bias (SEEG-RFTC group: *P* = 0.20). The sample size of the MRgLiTT group is <10.

#### 3.3.2. Subgroup analysis

**Figure 4:**
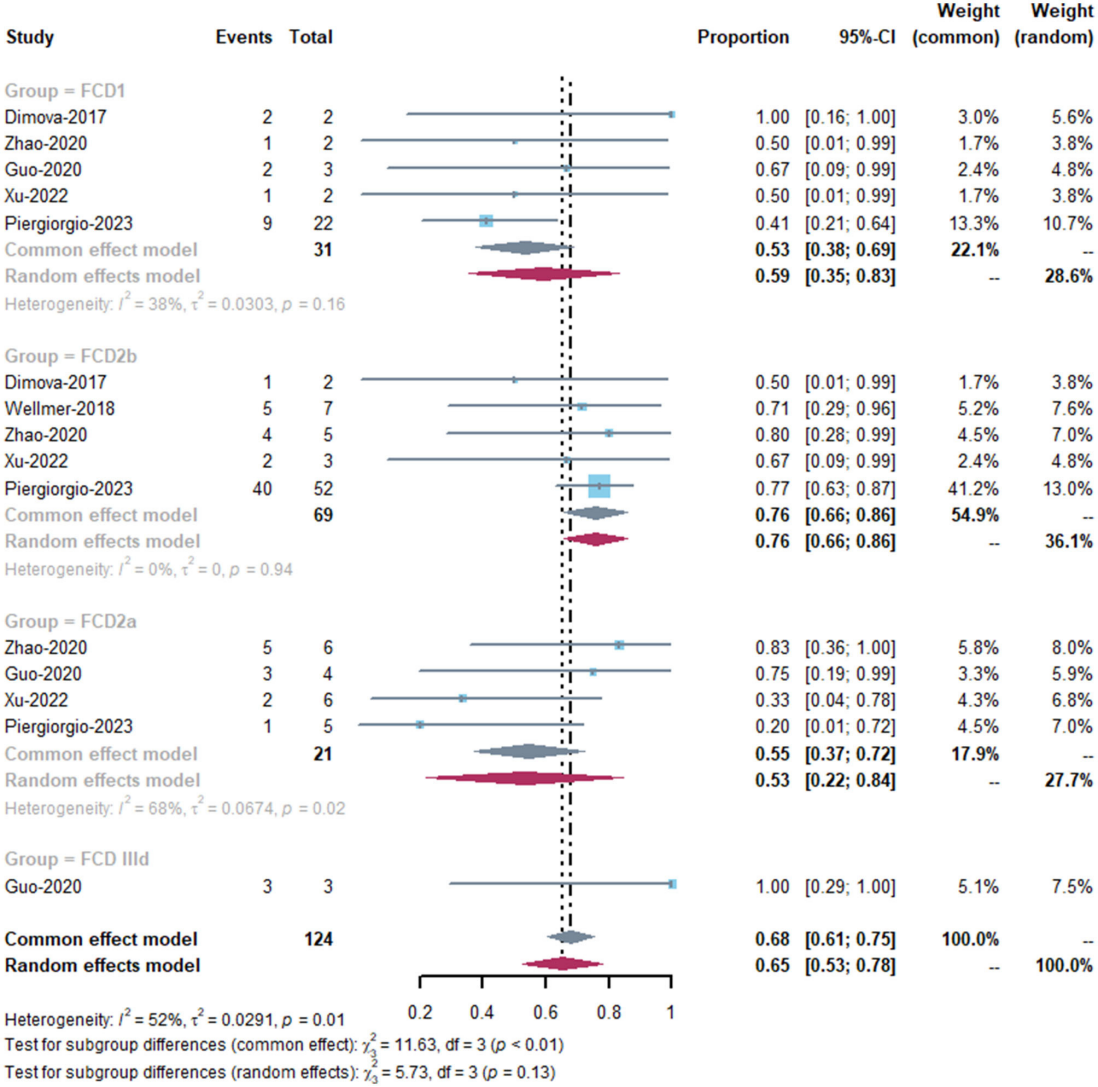
The subgroups of SEEG-RFTC in different type of FCD.

As there was no specific classification of FCD patients in the MRgLiTT group and the heterogeneity of the MRgLiTT group was low (0%), we performed subgroup analysis mainly in the SEEG-RFTC group. There were significant differences in the seizure-freedom rate among the three subtypes FCD1, FCD2a, and FCD2b (*P* < 0.01) (Figure 4). Patients with FCD2b had better response to SEEG-RFTC than FCD1 and FCD2a patients (*P* < 0.01).

In addition, we evaluated age and gender as a factor for heterogeneity. The results showed that age (>18 vs. ≤ 18; MRgLiTT: *P* = 0.73; SEEG-RFTC: *P* = 0.83) and sex (male vs. female; MRgLiTT: *P* = 0.73; SEEG-RFTC: *P* = 0.83) were not influential factors for epilepsy freedom in FCD patients in either the MRgLiTT or the SEEG-RFTC groups.

### 3.4. Complications

**Figure 5:**
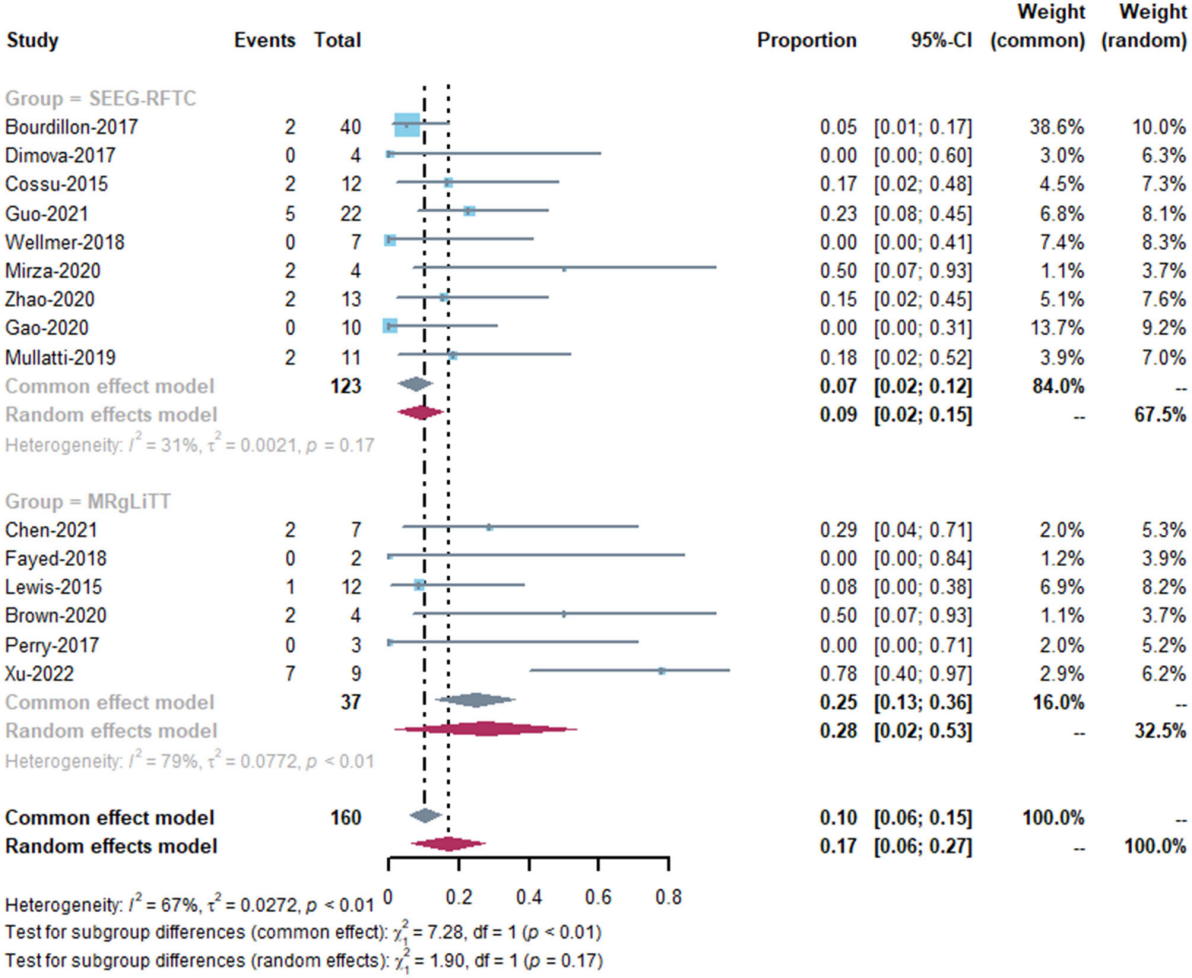
The complication rate of MRgLiTT and SEEG-RFTC.

Fourteen studies reported postoperative complication rates after MRgLiTT or SEEG-RFTC (15, 17, 19–24, 26–31) (Table 2). The overall complication rate was 17.1% (27/150), with a rate of 11.9% (19/159) for transient complications, 3.1% (5/159) for long-term complications, and 2% (3/159) for complications that could not be classified due to lack of information (two cases of visual impairment and one case of intraventricular hemorrhage + aseptic meningitis). In the MRgLiTT group, postoperative complications occurred in five patients (32.4%; 12/37), whereas in the SEEG-RFTC group, 15 patients (12.3%; 15/122) had postoperative complications. However, there was no significant difference in the complication rate in FCD patients between the two surgical methods (*P* = 0.62) (Figure 5). There were also no significant differences between the two groups in either transient (*P* = 0.63) or permanent complications (*P* = 0.53). There were four serious complications reported (2.7%, 4/150), including hemiparesis in two patients, focal hemorrhage with left leg paralysis in one patient, and ventricular hemorrhage with aseptic meningitis in one patient.

**Table 2:** Complications in FCD patients.

	**Transient**	**Long-term**	**Unable to categorize**
Bourdillon et al. (26)	Partial aphasia	Hand palsy	
Dimova et al. (29)	0	0	
Cossu et al. (28)	Motor deficit of the left foot	Dense right hemiparesis	
Guo et al. (30)	Slow speed, 3^*^muscle weakness	Hemiparesis	
Wellmer et al. (17)	0	0	
Mirza et al. (35)		Hemorrhage + left leg paresis, visual disturbance and dysarthria	
Zhao et al. (36)	2^*^Pneumocephalus		
Gao et al. (37)	0	0	
Mullatti et al. (38)	Transient R hand deficit, Transient L motor deficit		
Chen et al. (21)	2^*^Contralateral limb weakness	0	
Fayed et al. (22)	0	0	
Lewis et al. (23)	0	0	Intraventricular hemorrhage + aseptic meningitis
Brown et al. [2020]	0	0	2^*^Visual impairment
Perry et al. (24)	0	0	
Hu et al. (25)	7^*^Transient limb numbness		

## 4. Discussion

This study was the first meta-analysis comparing the efficacy and safety of MRgLiTT vs. SEEG-RFTC in patients with epilepsy caused by FCD. Patients with other diseases and those who failed to respond in prior surgeries were excluded. To reduce the risk of bias, we excluded case reports and literature employing fewer than five patients to reduce the bias due to patient selection. In some articles, there were patients with multiple treatments for multiple lesions, and we counted the number of patients according to the number of procedures (23, 29).

According to this systematic review, there was no significant difference in the efficacy between MRgLiTT and SEEG-RFTC in patients with FCD. In 2021, a meta-analysis comparing the two procedures in all drug-resistant epilepsy showed a significant difference in the postoperative rate of seizure freedom between patients receiving MRgLiTT (65%; 95% CI 56–74%) and those receiving SEEG-RFTC (23%; 95% CI 10–39%) (*P* = 0.00). The discrepancy may be due to the different sizes of ablation lesions. MRgLiTT-induced lesions were often greater than those caused by SEEG-RFTC. The MRgLiTT treatment technique can be repeated as needed to achieve overlapping thermal ablation. In this meta-analysis, the included FCD patients had a more limited range of lesions; thus, the difference was not detected. These findings suggest that the surgical choices for patients with FCD are more varied.

The heterogeneity in the SEEG-RFTC group was very high (*I*^2^ = 91%). However, the different FCD subtypes may be responsible for the increased heterogeneity. In our subgroup analysis, SEEG-RFTC was shown to be significantly more effective in patients with the FCD2b subtype than in patients with other types of FCD. In addition, the time of surgery may be another factor contributing to the heterogeneity. We found that the results of efficacy reported in two articles (26, 28) that included FCD patients receiving SEEG-RFTC at the earliest times (2008–2013, 2003–2013) (0/11, 6/40) differed significantly from those reported by the other articles. This may be attributed to the lack of accurate patient assessment and the unskilled use of the treatment. After excluding these two articles, SEEG-RFTC achieved a cure rate of 69.8% (127/182) for epilepsy in patients with FCD that is significantly higher than MRgLITT (*P* < 0.01). It means that SEEG-RFTC is currently a better treatment option for patients with FCD compared to MRgLITT.

Both MRgLiTT and SEEG-RFTC are considered safe treatments. Transient somatosensory and motor dysfunction is the most common postoperative complication for both MRgLiTT and SEEG-RFTC (40, 40%), which may be due to the local edema caused by the adjacent brain areas. The dysfunction also recovered as the edema subsided. It is important to note that both surgical approaches may cause severe bleeding and require extensive attention in clinical management. In addition, other meta-analyses have also shown a lower incidence of postoperative complications with MRgLiTT and SEEG-RFTC (2, 39, 40).

MRgLiTT and SEEG-RFTC do not show better treatment outcomes or have lower complication rates compared to surgical resection. In a study that included 2,014 patients who underwent FCD resection, the overall incidence of seizure freedom (Engel Class I) after surgical treatment was 55.8 ± 16.2%, showing comparable performance to the two approaches we studied (41). In a retrospective study in 2019, the rate of complications after surgical treatment of FCD was only 9% (17/188), which is also consistent with our data (1). However, compared to surgical resection, both MRgLiTT and SEEG-RFTC are minimally invasive and still allow for secondary surgery if the postoperative outcome is unsatisfactory (42, 43). In addition, a meta-analysis in 2019 showed that histological FCD type I, incomplete resection, and extratemporal location are factors for recurrence after patients receiving epilepsy surgery for FCD (44). Additional patient data are needed to explore whether these FCD patients with poor surgical outcomes can benefit from MRgLiTT and SEEG-RFTC.

Our results suggest that both MRgLiTT and SEEG-RFTC are effective and safe treatments for patients with FCD. This is the first study to compare the efficacy and prognosis between MRgLiTT and SEEG-RFTC in patients with FCD. Our results show that there is no significant difference in the efficacy and prognosis between MRgLiTT and SEEG-RFTC. This result suggests multiple options for the treatment of patients with FCD. In this study, postoperative complications were analyzed uniformly in all patients, but in fact, different complications may have different prognoses in patients. Therefore, we recalculated permanent complications (defined as complications that require long-term treatment or have a long-term impact on the patient's quality of life). There were no serious complications in the MRgLiTT group, and five serious complications were reported in the SEEG-RFTC group. However, this lacked statistical significance due to the small number of patients.

Our study has several limitations. (1) The different inclusion criteria among the included studies led to differential variation in patient selection, resulting in heterogeneity. (2) The length of follow-up varied among the studies, and the seizure-suppression effect of surgery may deteriorate with the extension of follow-up. (3) The small number of patients undergoing MRgLiTT surgery and the lack of categorical information prevented subgroup analysis. (4) Almost all patients with FCD received MRgLiTT and SEEG-RFTC operation without pathological confirmation of the proposed diagnosis of FCD. However, although the exact type of pathology of the patient could not be confirmed, the results of this article may provide guidance on the surgical approach for patients with a proposed diagnosis of FCD. (5) Our study simply compared the effectiveness and safety of the two approaches, without considering the cost-benefit ratio and time of proficiency of new technologies. A comparison of these aspects could be considered in future studies.

## 5. Conclusion

In conclusion, both MRgLiTT and SEEG-RFTC are currently safe and effective minimally invasive procedures for patients with FCD. Subgroup analysis further revealed that SEEG-RFTC is significantly more effective for FCD Type 2b than other FCD types, suggesting that SEEG-RFTC is the optimal choice for this type of patients. Our conclusions are based on limited retrospective analysis, and randomized controlled trials are still needed to validate the conclusions.

## Data availability statement

The original contributions presented in the study are included in the article/Supplementary material, further inquiries can be directed to the corresponding author.

## Author contributions

YL proposed the study purpose. Searching strategy was developed and revised by JG and ZY. The revision and guidance of the article are carried out by MJ. All authors have contributed to the revision of the manuscript.
