# Supplementary figures and images for: Magnetic resonance-guided laser interstitial thermal therapy vs. stereoelectroencephalography-guided radiofrequency thermocoagulation in epilepsy patients with focal cortical dysplasia: a systematic review and meta-analysis
Source: Front Neurol. 2023 Oct 20;14:1241763. doi: 10.3389/fneur.2023.1241763 (PMC10625445; doi:10.3389/fneur.2023.1241763)

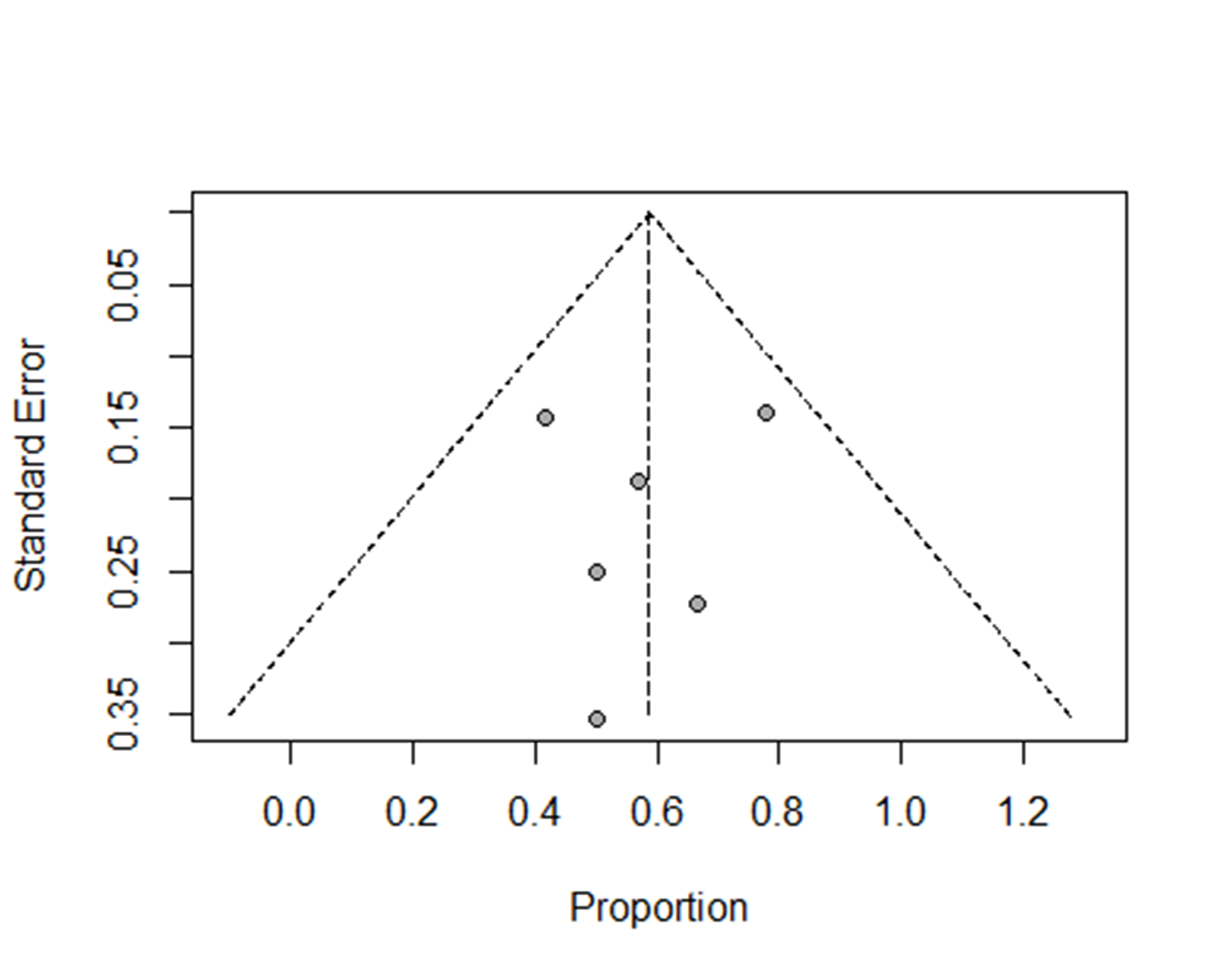

Supplement: Supplementary file 1 [file Image_1.TIF]

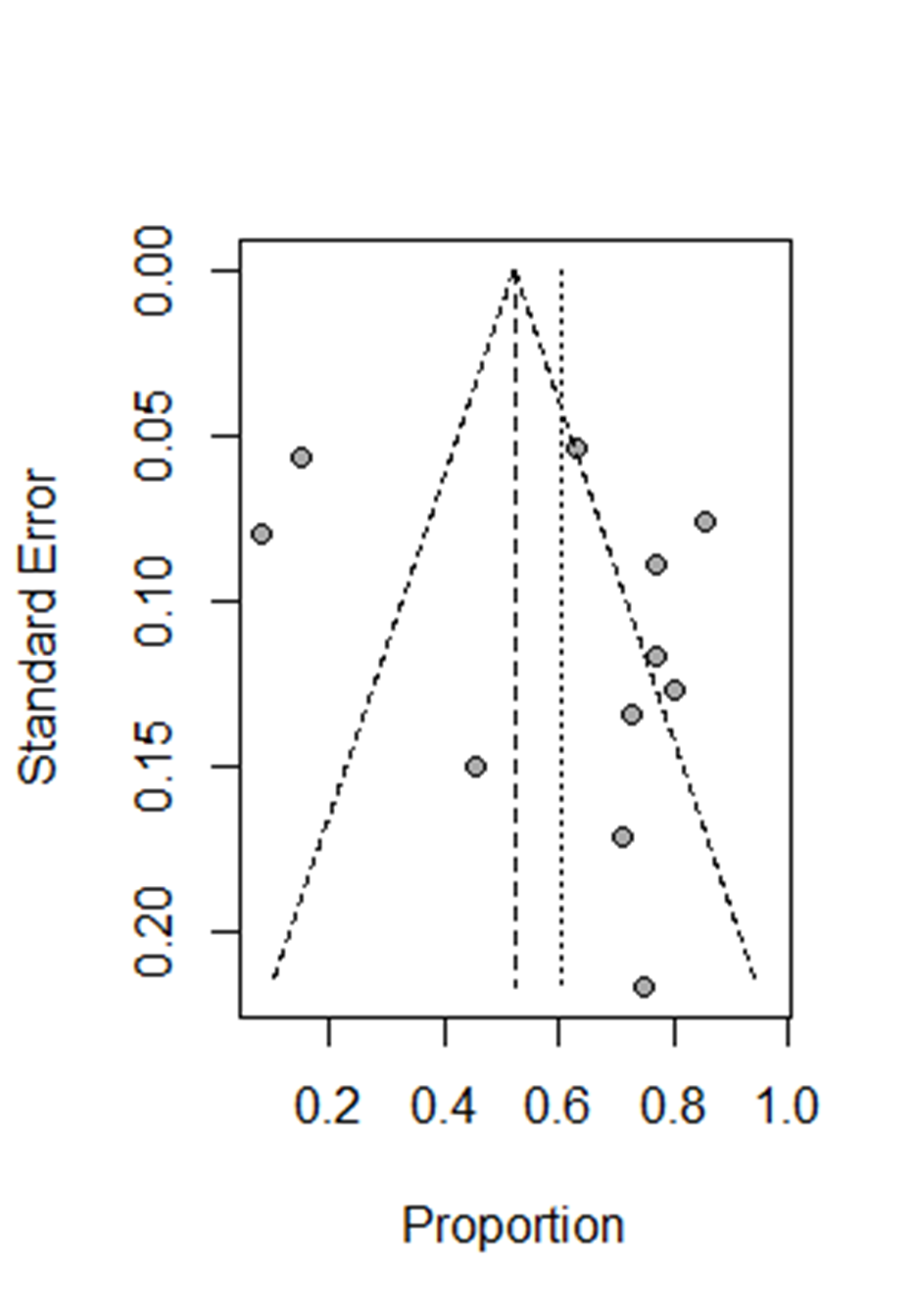

Supplement: Supplementary file 2 [file Image_2.TIF]
